# Effects of cardiac motion on dose distribution during stereotactic arrhythmia radioablation treatment: A simulation and phantom study

**DOI:** 10.1002/acm2.70021

**Published:** 2025-02-25

**Authors:** Takayuki Miyachi, Takeshi Kamomae, Fumitaka Kawabata, Kuniyasu Okudaira, Mariko Kawamura, Shunichi Ishihara, Shinji Naganawa

**Affiliations:** ^1^ Department of Radiology Nagoya University Graduate School of Medicine Nagoya Aichi Japan; ^2^ Radioisotope Research Center Nagoya University Nagoya Aichi Japan; ^3^ Department of Radiological Technology Nagoya University Hospital Nagoya Aichi Japan

**Keywords:** cardiac motion, CyberKnife, radioablation, stereotactic radiotherapy

## Abstract

**Purpose:**

Cardiac motion may degrade dose distribution during stereotactic arrhythmia radioablation using the CyberKnife system, a robotic radiosurgery system. This study evaluated the dose distribution changes using a self‐made cardiac dynamic platform that mimics cardiac motion.

**Methods:**

The cardiac dynamic platform was operated with amplitudes of 5 and 3.5 mm along the superior–inferior (SI) and left–right (LR) directions, respectively. The respiratory motion tracking of the CyberKnife system was applied when respiratory motion, simulated using a commercial platform, was introduced. The accuracy of respiratory motion tracking was evaluated by the correlation error between infrared markers and a fiducial marker. The dose distribution was compared with and without cardiac motion. The evaluations included error in the centroid analysis of the irradiated dose distribution, dose profile analysis in the SI and LR directions, and dose distribution analysis comparing the irradiated and planned dose distributions.

**Results:**

Cardiac motion increased the correlation error in the direction of motion. Cardiac motion displaced the centroid by up to 0.23 and 0.19 mm in the SI and LR directions, respectively. Cardiac motion blurring caused the distance of the isodose lines to become smaller (bigger) at higher (lower) doses in the SI direction. The gamma pass rate was reduced by cardiac motion but exceeded 94.1% with 1 mm/3% for all conditions. Respiratory motion tracking was also effective under cardiac motion. The cardiac motion slightly varied the dose at the edges of the irradiation volume.

**Conclusion:**

While cardiac motion increased respiratory tracking correlation errors, its effects on dose distribution were limited in this study. Further studies using motion phantoms that are close to a human or individual patient are necessary for a more detailed understanding of the effects of cardiac motion.

## 1 INTRODUCTION

Ventricular tachycardia (VT) is a severe arrhythmia that can be fatal. Antiarrhythmic drugs are often used to treat VT; however, they are not always effective in preventing arrhythmias. Although an implantable cardioverter‐defibrillator (ICD) is often used to prevent sudden cardiac death, frequent VT attacks and the consequent electroshocks required to treat them have been reported to worsen patients’ quality of life and long‐term prognosis.^1^, ^2^ Radiofrequency catheter ablation is a standard method for homogenizing arrhythmogenic substrates. In this therapy, focused pulses of radiofrequency energy are delivered directly to arrhythmogenic substrates from the endocardial side. However, 30%–50% of arrhythmias cannot be controlled by existing treatments, and an alternative option is needed.^3^, ^4^, ^5^

Currently, certain institutions have reported the use of radiation therapy systems to deliver high doses of stereotactic radiation to arrhythmogenic substrates,^6^, ^7^, ^8^ and this treatment is termed stereotactic arrhythmia radioablation.^9^, ^10^ Stereotactic arrhythmia radioablation is noninvasive and can access locations unreachable by radiofrequency catheter ablation.

Patient physiological motion during treatment adversely affects the dose distribution in high‐precision radiation therapy.^11^, ^12^ In stereotactic arrhythmia radioablation treatment, the heart is the irradiated target, and the dose distribution can be affected by cardiac and respiratory motion. Some radiation therapy systems are equipped with functions that monitor and immediately compensate for the physiological motion during treatment.^13^, ^14^ The CyberKnife system, which is a robotic radiosurgery system and dedicated treatment system for stereotactic radiosurgery (SRS) and stereotactic radiotherapy (SRT), also has a respiratory motion tracking system. However, as these current motion compensation methods focus on respiratory motion, they are unsuitable for cardiac motion.

A previous study^15^ showed that the means (and ranges) of target motion by cardiac motion alone in VT patients were 4.1 (1.4–8.0) mm in the superior–inferior (SI), 3.4 (1.0–4.8) mm in the left–right (LR), and 4.3 (2.6–6.5) mm in the anterior–posterior (AP) directions. Another study^16^ showed that the means (and ranges) of ICD lead motion were 5.0 (3.2–7.3), 3.4 (2.0–6.8), and 3.1 (2.1–4.9) mm in the SI, LR, and AP directions, respectively, although the motion may be attributed to both cardiac and respiratory motion. To the best of our knowledge, the effects of cardiac motion on the dose distribution in stereotactic arrhythmia radioablation of the CyberKnife system are yet to be thoroughly investigated. Thus, this study constructed a motion system that simulated cardiac motion. Consequently, the effects of cardiac motion on respiratory tracking and dose distribution were verified to assess the feasibility of stereotactic arrhythmia radioablation treatment under cardiac motion. This study will facilitate precise administration of radiation dose and thus enabling optimized radioablation therapy.

## 2 MATERIALS AND METHODS

### 2.1 Cardiac motion

**FIGURE 1:**
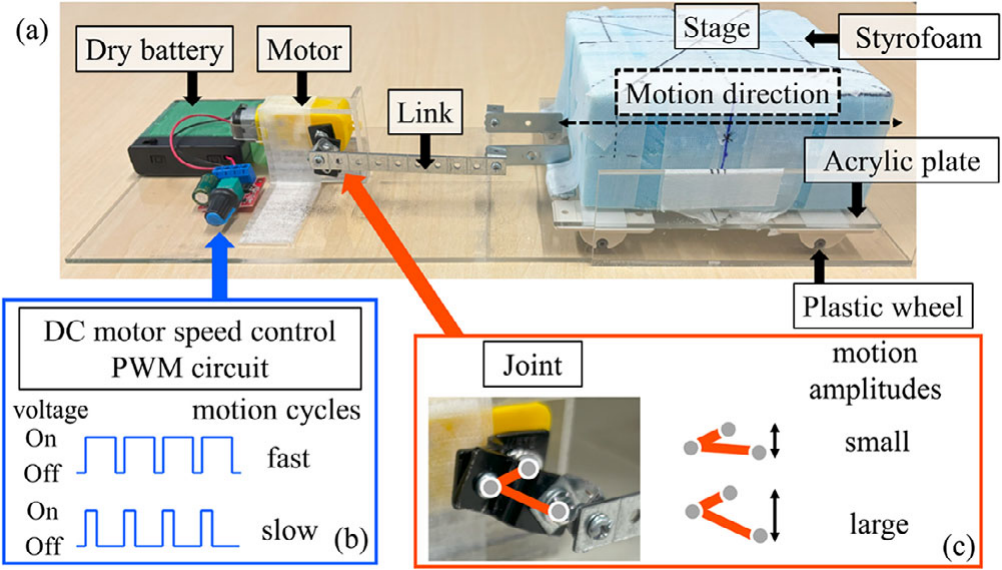
Overview of the self‐made cardiac dynamic platform used in this study. (a) Side view of the platform. (b) Method of adjusting motion cycles. A direct current (DC) motor speed control pulse width modulation (PWM) circuit could adjust the duty cycle and modify the rotation speed (motion cycles) of a motor (fast or slow). (c) Method of adjusting motion amplitudes. Adjusting the angle of a joint could modify the motion amplitudes (small or large).

A cardiac dynamic platform was developed to evaluate the effects of cardiac motion on dose distribution. This platform (Figure 1) comprised a stage on which the measuring phantom could be mounted. The stage, composed of a 60 mm thick styrofoam and a 3 mm thick acrylic plate with plastic wheels, could be moved back and forth in one direction. A metal link was fixed to the stage, and the other end of the link was connected to a joint to convert the motor rotation into a stage reciprocating linear movement. A direct current (DC) motor was powered by four dry cell batteries. A DC motor speed control pulse width modulation (PWM) circuit was placed between the batteries and the DC motor to adjust the motion cycles. The motion amplitudes could be set by adjusting the joint angle. The overall size of the cardiac dynamic platform measured 40, 15.6, and 8.5 cm in length, width, and height, respectively. Two types of cardiac motion [one‐dimensional (1D) and two‐dimensional (2D) motion] were simulated in this study. The amplitudes for each cardiac motion were based on the mean values of target and ICD lead motion from previous studies.^15^, ^16^ To simulate 1D cardiac motion in the SI direction, the platform was placed parallel to the SI direction (1D cardiac motion layout). The cardiac motion amplitude was 5 mm in the SI direction. 2D cardiac motion in the SI and LR directions was simulated by inclining the platform by 35° to the right from the SI direction (2D cardiac motion layout). The cardiac motion amplitudes were 5 and 3.5 mm in the SI and LR directions, respectively.^15^, ^16^ A sine wave was used for the cardiac motion. The cardiac motion cycle was set to 1 s.

### 2.2 Respiratory motion

The CIRS dynamic platform (Model 008PL; Computerized Imaging Reference System Inc., Norfolk, Virginia, USA) was used to simulate respiratory motion. This commercial platform is made of rigid low‐density plastic and contains both main and surrogate platforms. The main platform on which the phantom was placed moved in the SI direction, and the surrogate platform simulated the chest wall motion in the AP direction. The dynamic platform was placed on a couch parallel to the SI direction. The following waveform^17^ was used for the respiratory motion:

(1) $$P\;\left(t\right)=P_{0}\;-A\;cos^{6}\left(\pi t/\tau -\phi \right),$$

where P(t) is the position at time t, $P_{0}$ is the position at exhale (maximum displacement in the superior direction), A is the motion amplitude, $P_{0}-A$ is the position at inhale (maximum displacement in the inferior direction), τ is the period of motion cycle, and ϕ is the initial phase. In this study, we used A = 20 mm,^18^ τ = 5 s, and ϕ = 0.

### 2.3 Phantom and platform placement

**FIGURE 2:**
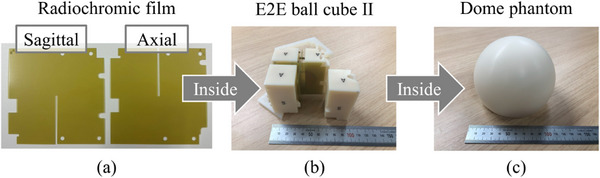
Set of measuring phantoms. (a) Radiochromic films were cut into special shapes. (b) Rectangular end‐to‐end (E2E) Ballcube II cassette in which a set of orthogonal films was inserted. (c) 12 cm diameter hemispherical dome phantom that enclosed the ballcube cassette.

**FIGURE 3:**
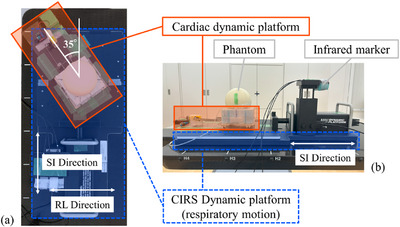
Overview of the platforms and phantoms placement. (a) Anterior side view of the 2D cardiac motion layout. The cardiac dynamic platform was inclined 35° to the right from the superior–inferior direction. (b) Right side view of the platforms and phantoms. The phantoms were placed on the stage of the cardiac dynamic platform. Infrared markers, which were used for respiratory motion tracking, were attached to the surrogate platform of the CIRS dynamic platform.

Figure 2 shows the details of the measuring phantoms used in this study. Using a laser cutter (VLS2.30; Universal Laser Systems, Scottsdale, Arizona, USA), radiochromic films (EBT3, Ashland Specialty Ingredients G.P., USA) measuring 63.5 mm × 63.5 mm with a 1‐mm slit were shaped, and they were inserted orthogonally into a cassette for the dose measurements. The rectangular end‐to‐end (E2E) Ballcube II cassette (ballcube; Accuray Inc., Sunnyvale, California, USA) had a 31.75 mm diameter high x‐ray attenuation acrylic spherical structure at the center and six metal markers that could be used for alignment. This cassette was inserted into a 12 cm diameter hemispherical dome phantom (Accuray Inc., Sunnyvale, California, USA). The phantom was placed on the cardiac dynamic platform stage (Figure 3). The cardiac dynamic platform was placed on the CIRS dynamic platform to simulate both the cardiac and respiratory motions. Figure 3a and b show the anterior and right side views of the 2D cardiac motion condition and phantom placement, respectively.

### 2.4 Radiation treatment system

The CyberKnife M6 Series system (version 11.1.3; Accuray Inc., Sunnyvale, California, USA) was used in this study. It comprises a compact 6 MV x‐ray linear accelerator mounted on a six‐joint robotic manipulator and an orthogonal kV x‐ray imaging system to obtain precise target location information. This system can irradiate an MV beam with high precision under continuous kV x‐ray image guidance. The Synchrony Respiratory Tracking System (Accuray Inc., Sunnyvale, California, USA) is a respiratory motion compensation system integrated into the CyberKnife system that estimates the target position in real time from the locations of infrared markers set on a patient's chest wall during treatment. The target locations at 15 time points are acquired using the kV x‐ray imaging system, and the positions of infrared markers are acquired simultaneously. The correlation model between the target motion and the infrared markers’ motion is automatically generated by the tracking system. By moving the six‐joint robotic manipulator using the correlation model, the CyberKnife system is capable of performing dynamic tracking irradiation while compensating for respiratory motion. The correlation model is generated in linear, quadratic, or zero‐quadratic approximations.^19^ The kV x‐ray imaging is repeated during treatment, and the correlation model is updated periodically considering the latest 15 time points. In this study, three time points were updated every 60 s. In conventional SRT, a small gold marker is often implanted as a fiducial marker near the target to improve visibility for identifying the target location on the kV x‐ray imaging system. In radioablation treatment, an ICD lead is often implanted near the target and can be considered an alternative to a gold marker.^20^, ^21^ In this study, an ICD lead (6935  M, Medtronic, Dublin, Ireland) was used as a fiducial marker for dynamic tracking irradiation and affixed to the surface of the dome phantom at 45° to the lower right. As a result, the ICD lead was placed at about 60 mm from the center of the cassette.

### 2.5 Treatment planning

A SOMATOM confidence scanner (Version: Syngo CT VB20A, Siemens Healthineers, Forchheim, Germany) was used to scan the phantom with 1D and 2D cardiac motion layouts, respectively. The CyberKnife system's treatment planning for respiratory tracking is typically performed using expiratory breath‐holding computed tomography (CT) to reduce blurring of a fiducial marker. In this study, the CT scans were performed with both platforms stationary. The CIRS dynamic platform was in the maximum expiratory position for respiratory motion. The cardiac dynamic platform was in the middle position for cardiac motion. The CT scan conditions were the following: tube voltage, 120 kVp; tube current, effective 250 mAs; and rotation time, 1.0 s. Image reconstruction was performed using the following parameters: slice thickness, 1.0 mm; field of view, 500 mm; matrix 512 × 512; and with iterative metal artifact reduction (iMAR). Treatment planning was performed using a Precision treatment system (Full Software Version 3.3.1.3 [2], Accuray Inc., Sunnyvale, California, USA) as treatment planning system (TPS). InCise2 micro multileaf collimators (MLC; Accuray Inc., Sunnyvale, California, USA) are used in stereotactic arrhythmia radioablation treatment. The CyberKnife system's E2E process using MLC is described in the CyberKnife's Physics Essential Guide^22^ supplied by the vendor, and this E2E process was applied in this study. Due to the specifications of the CyberKnife system, it was necessary to create an E2E plan for each condition, such as tracking methods and CT data. Specifically, a no‐motion tracking plan for 1D cardiac motion layout was created, while respiratory motion tracking plans for 1D and 2D cardiac motion layouts, respectively, were created. The ICD lead was selected for use as a fiducial marker. The plans were created using sequential/isocentric optimization, and the dose calculation was performed with the finite size pencil beam algorithm. The plans were created to deliver a dose of 420 cGy (70% isodose line) to the planning target volume (PTV), which was an area 3 mm inside the 31.75 mm diameter high x‐ray attenuation acrylic spherical structure of the ballcube.^22^ The dose distribution was spherical and centered on the geometric center of the PTV. Fifty to fifty‐eight non‐coplanar beams were used to form the spherical dose distribution. The maximum PTV dose was 600 cGy, and the coverage was in the range 99.76%–99.86%.

### 2.6 Irradiation

The platforms and phantoms were placed on the couch of the CyberKnife system, and infrared markers were fixed on the surrogate platform of the CIRS dynamic platform (Figure 3b). Six metal markers in the ballcube were used for six‐axis alignment correction. Alignment was performed in the maximum expiratory position for respiratory motion, and in the middle position for cardiac motion. After alignment, the no‐motion tracking plan was performed with and without cardiac motion, respectively. The respiratory motion tracking plan was performed under respiratory motion with and without cardiac motion, respectively. Moreover, these irradiations were performed three times under each condition.

### 2.7 Analysis

To evaluate the accuracy and reproducibility of the cardiac platform performance, an Artis Q biplane (Version: VD12A 230615; Siemens Healthineers, Forchheim, Germany) was used to acquire continuous fluoroscopic images.^23^ Spatial calibration was performed at the isocenter position with a pixel resolution of 0.096 mm/pixel. Spatial calibration accuracy was verified by measuring a metal sphere of known size (41.30 mm) at the isocenter position five times. A high‐density 1.5 mm marker was placed on the stage of the cardiac dynamic platform with the measuring phantoms. The marker was placed at the isocenter position and motion was evaluated in 1D and 2D cardiac motion layouts. The platform was placed and scanned five times in each layout. The center of mass coordinates of the marker was calculated from each frame though binarization using a python script in a Jupyter Notebook (Version 6.4.5) on an Anaconda Navigator (Version 2.1.4, Anaconda Inc., USA), thus providing the platform position as a function of time. Root mean square error (RMSE) was calculated from the difference between the position of the measured platform and the ideal sine motion in all frames for each condition. The mean RMSE was calculated from five measurements under the same conditions. The fluoroscopic conditions were set as follows: cine rate, 15 frames per second (66.7 ms); number of frames, 100 frames; tube voltage, 77 kVp; tube current, 40 mA; and average pulse width, 3.5 ms.

To evaluate the tracking accuracy of the CyberKnife system, correlation errors were evaluated. The correlation error was the difference between the predicted target position calculated from the correlation model and the actual target position detected in the x‐ray images. The correlation errors were automatically calculated by the CyberKnife system, updated with periodic updates of the correlation model and saved to a log file.

Three types of analyses were performed on the irradiated films: (1) error in the centroid analysis of the irradiated dose distribution, (2) dose profile analysis in the SI and LR directions, and (3) dose distribution analysis comparing the irradiated and planned dose distributions. The films were scanned 24 h post exposure using an EPSON EXPRESSION 10000 XL scanner (Epson Seiko Corporation, Japan). The image type was set to 48‐bit color, and the resolution was set to 300 dpi for the centroid analysis and 75 dpi for the dose profile and dose distribution analyses. The red channel was selected for the analysis. A dose calibration curve was created from 0 to 1000 cGy using 15 data points. The dose was delivered to each film using the same CyberKnife system. These film pieces were scanned using the same film scanner and technique as for the analysis films.

**FIGURE 4:**
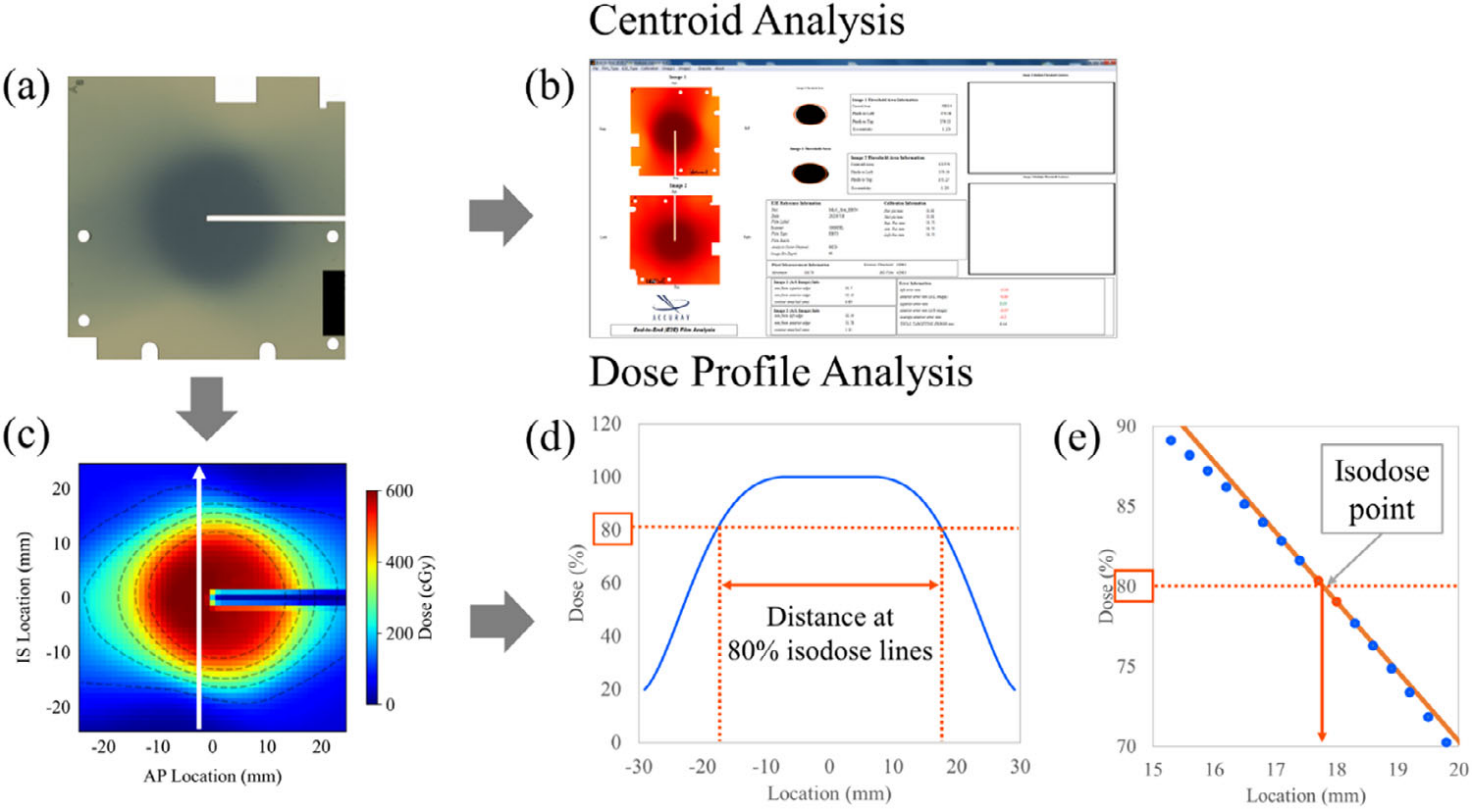
Methods of analysis. (a) and (b): Centroid analysis. (a), (c), (d), and (e): Dose profile analysis. (a) An irradiated film. (b) An end‐to‐end (E2E) Film Analysis software. This software can measure the error in the centroid from the difference between the centroid of the thresholded irradiation distribution and the film center which is same to the centroid of planned distribution. (c) A cropped and dose‐transformed distribution from the irradiated film. The white solid arrow indicates the location 3 mm away from the film center slit, where the dose profile was measured. (d) A distance between 80% isodose lines shown on the dose profile (the blue solid line). (e) An isodose point. This point was calculated using a linear approximation from two points (the red points) adjacent to the 80% isodose. The distance between 80% isodose line was calculated from this isodose point and another isodose point.

Centroid analysis was performed for a pair of orthogonal films using an E2E Film Analysis software (version 4.0, Accuray Inc., Sunnyvale, California, USA), which is used in the CyberKnife system's QA check (Figure 4b). The error in the centroid of the dose distribution was calculated from the distance between the centroid of the thresholded irradiation distribution and the film center, which is same as to the centroid of planned distribution. These values included all uncertainties throughout the treatment process. To evaluate the effect of cardiac motion on the centroid of the dose distribution, the displacement values of the centroid were obtained by subtracting the value without cardiac motion from that with cardiac motion.

The SNC Patient software (version 6.2.3.5713; SUN NUCLEAR Corporation, Melbourne, Florida, USA) was used to convert film optical densities to doses. Dose profile and distribution analyses were performed using the dose data converted by this software.

Dose profile analysis was performed in the SI and RL directions, 3 mm away from the film center slit, to eliminate the influence of the film edges (Figure 4c). The distances between isodose lines were calculated from isodose points at both ends of the spherical dose distribution in a certain direction (Figure 4d). Each isodose point was calculated using a linear approximation from two points adjacent to the specific dose (Figure 4e), because the dose values were obtained at intervals of approximately 0.34 mm (75 dpi). The distances between the isodose lines were calculated for the 90%, 80%, 70%, 50%, and 20% isodoses, respectively.

Dose distribution analysis was performed by comparing the irradiated dose distribution with the calculated dose distribution of the treatment planning. The gamma pass rate was used as an evaluation index. The gamma pass rate was calculated using an open‐source code PyMedPhys software^24^ (Version 0.39.3) in a Jupyter Notebook on an Anaconda Navigator. The calculation was performed over a 25 mm × 25 mm central area of film. Data within 2 mm from the film slit was omitted to remove the effects of the film shape. The gamma pass rate was calculated using the distance to agreement (DTA) and dose difference (DD) criteria of 2 mm/3%, 2 mm/2%, or 1 mm/3% with global normalization. The threshold cutoff percentage (threshold) was set at 10%.

In the three analyses, the mean and standard deviation (SD) were calculated considering the results of three repetitions under the same conditions.

## 3 RESULTS

### 3.1 Cardiac platform performance

The spatial resolution calculated from the metal sphere of known size was 0.096 mm/pixel, which is the same as the calibrated spatial resolution. The same value was obtained in five repeated measurements. The mean ± 1SD of the RMSEs were 0.50 ± 0.09  and 0.01 ± 0.00 mm in the SI and RL directions, respectively, in the 1D cardiac motion layout. The mean ± 1SD of the RMSEs were 0.73 ± 0.15  and 0.51 ± 0.11 mm in the SI and RL directions, respectively, in the 2D cardiac motion layout. When the mean ± 1SD of the amplitudes of sine waveforms were set to 5.2 ± 0.1 mm in the SI direction in the 1D cardiac motion layout and 5.8 ± 0.1  and 4.1 ± 0.1 mm in the SI and RL directions, respectively, in the 2D cardiac motion layout, the peak‐to‐valley distances of the measured waveforms matched the amplitudes of sine waveforms.

### 3.2 Correlation model analysis

**FIGURE 5:**
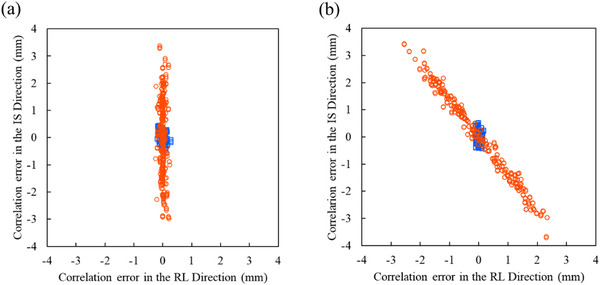
Correlation errors in the superior–inferior and left–right directions. (a) and (b) Correlation errors in 1D cardiac motion layout and 2D cardiac motion layout, respectively. The blue square plots indicate the correlation errors without cardiac motion. The red circle plots indicate the correlation errors with cardiac motion. These plots show the correlation errors for all model points of three repetitions under same conditions.

Figure 5 shows the correlation errors for all the model points of three repetitions in the SI and LR directions. A positive correlation error in the SI direction (LR direction) indicates that the target position in the x‐ray images is further to the superior (to the left) than the target position calculated from the correlation model. Table S1 presents the mean, maximum, and minimum values of the correlation errors. Cardiac motion did not change the mean values of the correlation errors, but it changed the maximum and minimum values of the correlation errors in the direction of cardiac motion. Cardiac motion changed the maximum (and minimum) values of the correlation errors in the 1D cardiac motion layout by 2.32 (−2.10), −0.01 (−0.08), and 0.07 (0.01) mm in the SI, LR, and AP directions, respectively, and in the 2D cardiac motion layout by 2.56 (−2.66), 2.01 (−2.25), and 0.14 (−0.16) mm in the SI, LR, and AP directions, respectively.

### 3.3 Error in the centroid analysis

Table 1 presents the error results from centroid analysis of the dose distribution. The mean errors of the centroid ranged from ‐0.45 to 0.59 mm under all conditions. Cardiac motion displaced the centroid by up to 0.23, 0.19, and 0.15 mm in the SI, LR, and AP directions, respectively, in all conditions.

**TABLE 1:** Error in the centroid analysis on each motion condition.

Respiratory motion			Off		On			
			1D		1D		2D	
Cardiac motion			Off	On	Off	On	Off	On
Error in the centroid (mm)	SI	Mean	−0.45	−0.22	−0.04	0.19	0.25	0.29
		Displacement	0.23		0.23		0.04	
	LR	Mean	0.16	0.12	0.00	0.09	0.21	0.40
		Displacement	−0.04		0.09		0.19	
	AP	Mean	−0.29	−0.14	0.09	0.12	−0.17	−0.32
		Displacement	0.15		0.03		−0.15	

The mean was calculated from the results of three replicates under the same conditions. The displacement was obtained by subtracting the value without cardiac motion from that with cardiac motion.

Abbreviations: 1D, one‐dimensional cardiac motion layout; 2D, two‐dimensional cardiac motion layout; AP, anterior–posterior; LR, left–right; SI, superior–inferior.

### 3.4 Dose profile analysis

**FIGURE 6:**
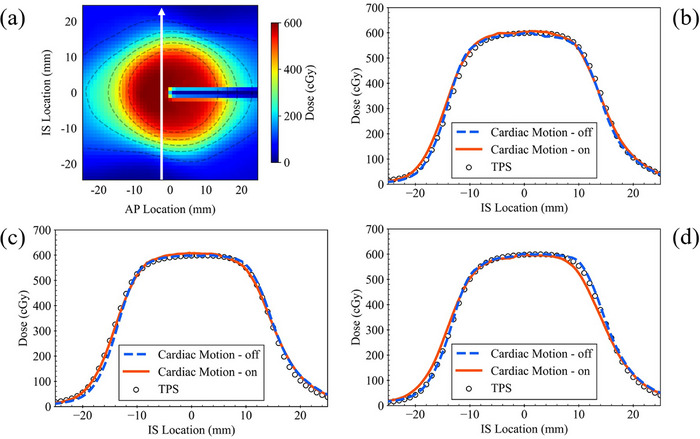
Dose profile analysis in the superior–inferior direction. (a) Analyzed location of the dose profile on dose distribution (the white arrow). (b), (c), and (d) Dose profile of no‐motion tracking in 1D cardiac motion layout, respiratory motion tracking in 1D, or 2D cardiac motion layout, respectively. The blue dotted line indicates the average of the profile without cardiac motion. The red solid line indicates the average of the profile with cardiac motion. The black no‐fill symbol indicates the dose profile calculated by the treatment planning system (TPS).

Figures 6 and S1 show the results of the dose profile analysis in the SI and LR directions, respectively. Figure 6a shows the analyzed location of the dose profile for each dose distribution, and Figure 6b, 6c, and 6d show the dose profiles of no‐motion tracking in 1D cardiac motion, respiratory motion tracking in 1D cardiac motion, and 2D cardiac motion, respectively. Tables S2 and S3 list the distances of the isodose lines in the SI and LR directions, respectively.

No distinct difference was observed between no‐motion and respiratory motion tracking. A slight difference in the profile was observed for respiratory motion tracking in the 2D cardiac motion (Figure 6d). In the SI direction, cardiac motion reduced the distance of the high isodose lines, and conversely increased the distance of low isodose lines.

### 3.5 Dose distribution analysis

Table 2 presents the results of the gamma pass rate for the irradiated dose distribution and the dose distribution calculated using the TPS. The gamma pass rate exceeded 99.9% with 2 mm/3% for all conditions. Even with 2 mm/2%, which is a stricter criterion, the gamma pass rate was greater than 96.9%. However, the gamma pass rate with 1 mm/3% in the sagittal plane of the respiratory motion tracking in 2D cardiac motion was 94.1%. This can be observed from the profile in the SI direction in Figure 6d, which shows that the doses were reduced at the edge of the superior of the target.

**TABLE 2:** Gamma pass rate on each motion condition.

	Respiratory motion					
	Off		On			
	Cardiac motion					
	1D		1D		2D	
Gamma pass rate (％) Mean ± 1SD	Off	On	Off	On	Off	On
2 mm/3%						
Axial	100 ± 0.0	100 ± 0.0	100 ± 0.0	100 ± 0.0	100 ± 0.0	99.7 ± 0.4
Sagittal	99.9 ± 0.1	100 ± 0.0	100 ± 0.0	100 ± 0.0	100 ± 0.0	99.3 ± 0.9
2 mm/2%						
Axial	100 ± 0.0	100 ± 0.0	99.6 ± 0.4	99.9 ± 0.1	100 ± 0.0	98.0 ± 2.1
Sagittal	98.7 ± 1.1	99.9 ± 0.2	100 ± 0.0	99.8 ± 0.4	99.3 ± 1.3	96.9 ± 3.0
1 mm/3%						
Axial	100 ± 0.0	100 ± 0.1	99.9 ± 0.2	100 ± 0.0	100 ± 0.0	98.8 ± 1.8
Sagittal	99.5 ± 0.5	99.4 ± 0.4	99.2 ± 1.4	97.7 ± 3.0	99.8 ± 0.4	94.1 ± 5.9

2 mm/3%, 2 mm/2%, and 1 mm/3% indicate the distance to agreement (DTA) and dose difference (DD) criteria. The mean and standard deviation (1SD) were calculated from the results of three replicates under the same conditions.

Abbreviations: 1D, one‐dimensional cardiac motion layout; 2D, two‐dimensional cardiac motion layout.

## 4 DISCUSSION

In this study, we utilized both the self‐made cardiac and CIRS dynamic motion platforms to reproduce cardiac and respiratory motions. This facilitated the separation of each motion and examination of their influence on the dose distribution. Although the measured amplitudes of cardiac motion in this study were larger than the setting amplitudes, the cardiac dynamic platform has sufficient accuracy to simulate the cardiac motion in this study because RMSEs were < 1 mm. Reproducibility is the most important for comparison, and the platform showed high reproducibility with the maximum SD of peak‐to‐valley distance being < 0.1 mm in five measurements.

Muacevic et al.^25^ conducted a phantom study for patients with lung cancer and reported that respiratory motion tracking improved the dose profile in the direction of motion. Respiratory motion tracking is a useful technology to prevent the distortion of dose distribution caused by respiratory motion. Radek et al.^20^ performed in vivo respiratory motion tracking using an ICD lead, which was an alternative to a metal marker, during SRT for VT patients. Respiratory motion tracking has also been required for SRT in the cardiac region, just as in other regions affected by respiratory motion. The presented study indicates that respiratory motion tracking using an ICD lead can reduce the negative effects of respiratory motion on dose distribution, even in situations involving both respiratory and cardiac motions.

Cardiac motion caused variations in the correlation errors of the model. Infrared markers’ motion corresponded to respiratory motion but did not correspond to cardiac motion. The phases of cardiac motion changed depending on the timing of the x‐ray imaging used for modeling. Cardiac motion made the relationship between the infrared marker position and the ICD lead position irregular. It caused irregularities in tracking modeling for respiratory periodic motion. The correlation errors tended to increase in the direction of cardiac motion. The dose distribution depends on the accuracy of the correlation model, and the correlation model errors lead to spatial deformation of the dose distribution.

The maximum difference in the E2E test must not exceed 0.95 mm, as described in the AAPM TG‐135 report.^26^ The results of the E2E test were within the tolerance range under all conditions. Notably, the analytical accuracy of the E2E test itself has been estimated to be approximately 0.3 mm,^27^ and must be considered when interpreting a difference of less than 0.3 mm. Even with a cardiac motion of 5 mm, which is the average patient cardiac motion, the displacement of the centroid is smaller than 0.3 mm; thus, cardiac motion does not cause displacement in the centroid of the dose distribution above detection accuracy. In this study, CT for treatment planning was performed at the center position of the cardiac motion range, and the cardiac motion was a condition wherein the phantom moved in a sine wave for the same distance from the center position to both ends. In this case, the residence time on both sides is the same, and no systematic error occurs. If a CT scan for treatment planning happens to be performed at a maximum diastole or systole phase, the heart during irradiation moves to one side from the cardiac position of CT images. This is different from the condition for this study, and it is necessary to check whether the centroid of the dose distribution shifts.

Bortfeld et al.^11^ stated that organ movement can blur dose distribution and cause spatial deformation of the dose distribution. In this study, because of cardiac motion, the distances of the isodose lines tended to become smaller and bigger at higher and lower doses, respectively, in the SI direction. This was attributed to blurring.

The AAPM TG‐218^28^ report suggests universal tolerance limits of ≥ 95% and action limits of ≥ 90% with 3%/2 mm and a 10% dose threshold for intensity‐modulated radiation therapy (IMRT). In SBRT, a more stringent gamma criteria is important for assessing the high dose gradient. In this study, 2 mm/2% and 1 mm/3% were added as gamma criteria. The results were above the action limits for all conditions. Therefore, cardiac motion in this study did not have an unacceptable negative effect on the dose distribution. However, in 2D cardiac motion, it is not possible to satisfy the tolerance limits with the stricter criterion of 1 mm/3%. This may be attributed to blurring and deformation of the dose distribution.

To compensate for the dose blurring caused by cardiac motion during radioablation treatment, a margin should be adopted. Neuwirth et al.^20^ set a PTV margin of 0 mm to reduce potential toxicity to normal tissues, and Lukas et al.^16^ added an additional 3 mm CTV–PTV margin after evaluating efficiency and toxicity. Currently, the guidelines for radioablation treatment are not well established. Regarding the respiratory motion, Van Herk et al.^29^ estimated an additional margin for the effects of respiratory motion from changes in the isodose line coordinates. They calculated an additional margin from the average distance between the isodose line coordinates without respiratory motion and the prescribed isodose line coordinates with respiratory motion. An 80% isodose line is often used as the prescription dose for radioablation treatment.^21^ In this study, the distance of the 80% isodose line reduced by 1.4 mm in the SI direction owing to the 2D cardiac motion. Thus, a margin of approximately 1.4 mm in the SI direction is required to compensate for the effects of cardiac motion. The adaptation of a margin is considered appropriate to ensure the dose at the edge of the irradiation volume under cardiac motion; however, the amount of margin needs to be added carefully considering other margins. In actual clinical practice, cardiac motion occurs in three dimensions, and further verification of the margin is required. Because there is an optimal margin for each patient including the accuracy of the correlation model creation, the irregularity of cardiac motion must be considered and verified in individual patients.

A limitation of this study is that the cardiac motion was only considered as a 2D horizontal movement; moreover, the positional relationship between the target and the ICD lead was constant. In a clinical patient, the positional relationship between the target and ICD becomes complicated owing to irregular cardiac motion. Motion tracking accuracy may worsen owing to the rotation and deformation of the target. The amount of cardiac motion in this study was based on the mean values from previous studies; certain patients have a larger amount of cardiac motion. Cardiac motion conditions vary from patient to patient. The effect of cardiac motion on the dose distribution and the appropriate margins to compensate for the inadequate dose distribution must be considered for individual patients through sufficient evaluation using a dedicated phantom for at least the first several patients until a certainty treatment protocol is established.

## 5 CONCLUSION

A cardiac dynamic platform was developed to measure dose distribution while accounting for cardiac motion effects. Under the conditions of this study, the respiratory motion tracking of the CyberKnife system remained feasible despite cardiac motion. Although cardiac motion increased the respiratory tracking correlation errors in the directions of cardiac motion, its effects on dose distribution were limited in this study (gamma pass rate ≥ 94.1% with 1 mm/3%). In stereotactic arrhythmia radioablation, the high‐dose gradient would be used around the organs at risk such as stomach and coronary artery, to meet the dose constraints. In these areas, the slight dose distortion induced by cardiac motion should be noted. Further studies using motion phantoms that are close to a human or individual patient are necessary for a more detailed understanding of the effects of cardiac motion.

## AUTHOR CONTRIBUTIONS

The conceptual design of the study was carried out by Takayuki Miyachi, Takeshi Kamomae, Fumitaka Kawabata, Kuniyasu Okudaira, Mariko Kawamura, Shunichi Ishihara, and Shinji Naganawa. Takayuki Miyachi and Takeshi Kamomae wrote the main manuscript. Takayuki Miyachi collected the data and performed the data analyses. All authors read and approved the final manuscript as submitted.

## CONFLICT OF INTEREST STATEMENT

The authors declare no conflicts of interest.

## Supporting information



Supporting Information
