# Supplementary material for: Effects of cardiac motion on dose distribution during stereotactic arrhythmia radioablation treatment: A simulation and phantom study
Source: J Appl Clin Med Phys. 2025 Feb 25;26(5):e70021. doi: 10.1002/acm2.70021 (PMC12059291; doi:10.1002/acm2.70021)
Supplement: Supplementary file 1 — Supporting Information [file ACM2-26-e70021-s001.docx]

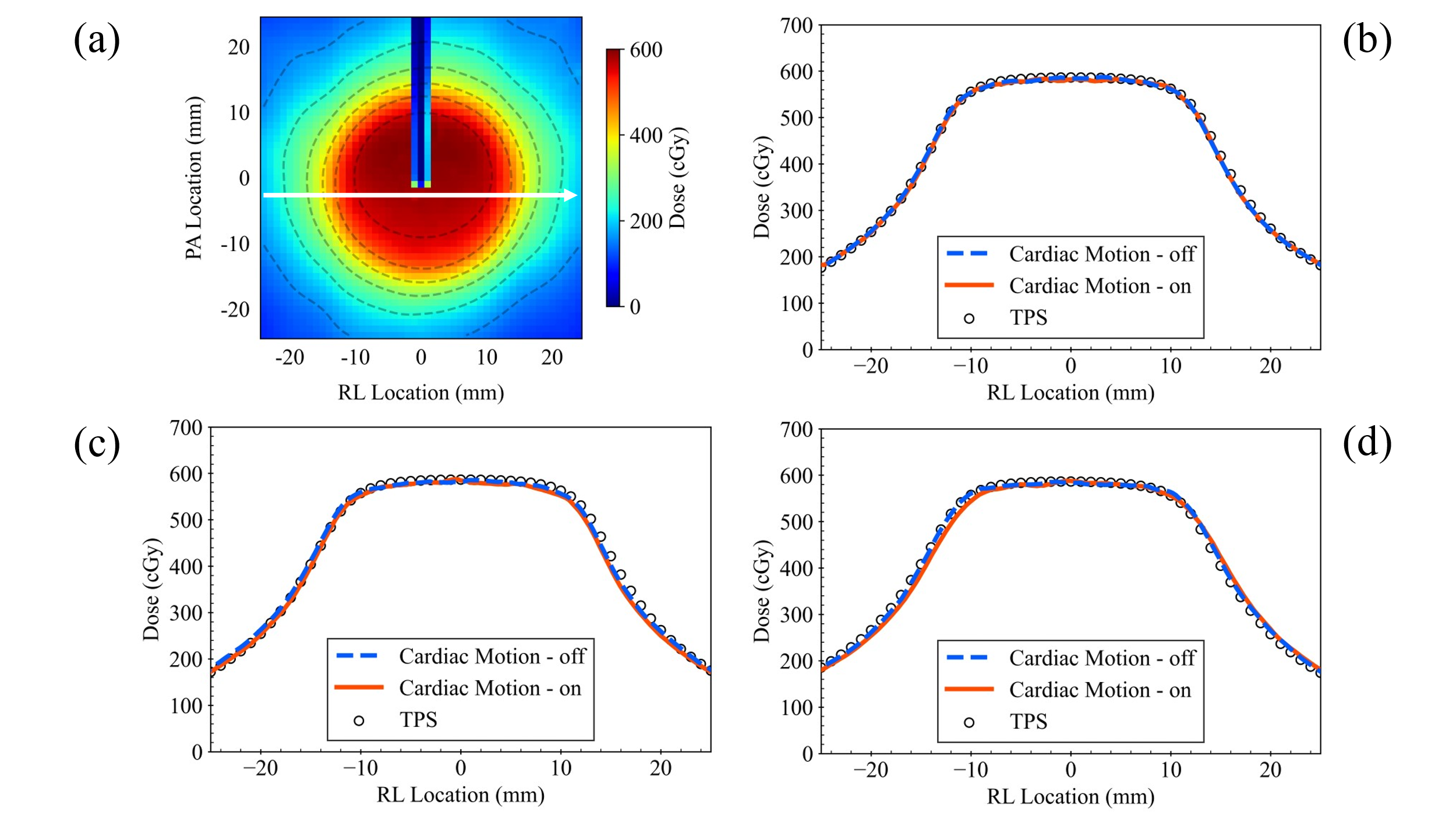


Fig. S1. Dose profile analysis in the left–right direction. (a) Analyzed location of the dose profile on dose distribution (the white arrow). (b), (c), and (d) Dose profile of no-motion tracking in 1D cardiac motion layout, respiratory motion tracking in 1D, or 2D cardiac motion layout, respectively. The blue dotted line indicates the average of the profile without cardiac motion. The red solid line indicates the average of the profile with cardiac motion. The black no-fill symbol indicates the dose profile calculated by the treatment planning system (TPS).

Table S1. Correlation errors on each cardiac motion

| Respiratory motion | | | On | | | |
| --- | --- | --- | --- | --- | --- | --- |
| Cardiac motion | | | 1D | | 2D | |
|  |  |  | Off | On | Off | On |
| Correlation error (mm) | SI | Mean | 0.00 | 0.00 | -0.01 | 0.07 |
|  |  | Maximum | 0.40 | 2.72 | 0.49 | 3.05 |
|  |  | Minimum | -0.44 | -2.54 | -0.43 | -3.09 |
|  | LR | Mean | 0.00 | 0.00 | 0.00 | -0.05 |
|  |  | Maximum | 0.15 | 0.14 | 0.10 | 2.11 |
|  |  | Minimum | -0.12 | -0.20 | -0.07 | -2.32 |
|  | AP | Mean | 0.00 | 0.00 | 0.00 | 0.01 |
|  |  | Maximum | 0.08 | 0.15 | 0.08 | 0.22 |
|  |  | Minimum | -0.10 | -0.09 | -0.09 | -0.25 |

The mean, maximum, and minimum were calculated from the results of three replicates under the same conditions. 1D: one-dimensional cardiac motion layout; 2D: two-dimensional cardiac motion layout; SI: superior–inferior, LR: left–right, AP: anterior–posterior.

Table S2. Distances between the same isodose lines along the superior–inferior direction on each motion condition.

| Distances at isodose lines (mm) | Respiratory motion | | | | | | |
| --- | --- | --- | --- | --- | --- | --- | --- |
|  | Off | | On | | | | |
|  | Cardiac motion | | | | | | |
|  | 1D | | | 1D | | 2D | |
|  | Off | On | | Off | On | Off | On |
| 90% |  | | | | | | |
| Mean ± 1SD | 19.6 ± 0.4 | 19.4 ± 0.4 | | 20.4 ± 0.4 | 19.5 ± 0.4 | 19.9 ± 0.4 | 17.9 ± 1.3 |
| Difference | -0.2 | | | -0.9 | | -2.0 | |
| 80% |  | | | | | | |
| Mean ± 1SD | 23.1 ± 0.1 | 22.8 ± 0.3 | | 23.5 ± 0.2 | 22.9 ± 0.4 | 23.3 ± 0.1 | 21.9 ± 0.5 |
| Difference | -0.3 | | | -0.6 | | -1.4 | |
| 70% |  | | | | | | |
| Mean ± 1SD | 25.3 ± 0.1 | 25.3 ± 0.2 | | 25.6 ± 0.2 | 25.4 ± 0.3 | 25.5 ± 0.1 | 24.6 ± 0.4 |
| Difference | 0.0 | | | -0.2 | | -0.9 | |
| 50% |  | | | | | | |
| Mean ± 1SD | 28.9 ± 0.0 | 29.5 ± 0.2 | | 29.3 ± 0.1 | 29.6 ± 0.1 | 29.2 ± 0.1 | 29.3 ± 0.3 |
| Difference | 0.6 | | | 0.3 | | 0.1 | |
| 20% |  | | | | | | |
| Mean ± 1SD | 36.5 ± 0.1 | 37.6 ± 0.1 | | 37.0 ± 0.1 | 37.8 ± 0.4 | 37.2 ± 0.1 | 38.0 ± 0.2 |
| Difference | 1.1 | | | 0.7 | | 0.8 | |

90%, 80%, 70%, 50%, and 20% indicate the specific isodose lines used for the calculation of the distances. The mean and standard deviation (1SD) were calculated from the results of three replicates under the same conditions. This difference was obtained by subtracting the value without cardiac motion from that with cardiac motion. 1D: one-dimensional cardiac motion layout; 2D: two-dimensional cardiac motion layout; SI: superior–inferior.

Table S3. Distances between the same isodose lines in the left–right direction on each motion condition.

| Distances at isodose lines (mm) | Respiratory motion | | | | | |
| --- | --- | --- | --- | --- | --- | --- |
|  | Off | | On | | | |
|  | Cardiac motion | | | | | |
|  | 1D | | 1D | | 2D | |
|  | Off | On | Off | On | Off | On |
| 90% |  | | | | | |
| Mean ± 1SD | 22.4 ± 0.1 | 22.1 ± 0.1 | 22.6 ± 0.0 | 21.9 ± 0.4 | 22.6 ± 0.4 | 21.2 ± 1.2 |
| Difference | -0.3 | | -0.8 | | -1.4 | |
| 80% |  | | | | | |
| Mean ± 1SD | 26.2 ± 0.2 | 26.2 ± 0.1 | 26.5 ± 0.1 | 26.0 ± 0.1 | 26.4 ± 0.2 | 25.9 ± 0.5 |
| Difference | 0.0 | | -0.5 | | -0.5 | |
| 70% |  | | | | | |
| Mean ± 1SD | 29.1 ± 0.1 | 29.0 ± 0.2 | 29.4 ± 0.1 | 28.9 ± 0.1 | 29.5 ± 0.2 | 29.2 ± 0.3 |
| Difference | -0.1 | | -0.5 | | -0.2 | |
| 50% |  | | | | | |
| Mean ± 1SD | 36.1 ± 0.1 | 36.1 ± 0.2 | 36.5 ± 0.2 | 35.8 ± 0.1 | 36.9 ± 0.1 | 36.5 ± 0.5 |
| Difference | -0.1 | | -0.7 | | -0.3 | |

90%, 80%, 70%, and 50% indicate the specific isodose lines used for the calculation of the distances. The mean and standard deviation (1SD) were calculated from the results of three replicates under the same conditions. This difference was obtained by subtracting the value without cardiac motion from that with cardiac motion. 1D: one-dimensional cardiac motion layout; 2D: two-dimensional cardiac motion layout; LR: left–right.
